# Lipophilic Triphenylphosphonium Cations Inhibit Mitochondrial Electron Transport Chain and Induce Mitochondrial Proton Leak

**DOI:** 10.1371/journal.pone.0121837

**Published:** 2015-04-30

**Authors:** Jan Trnka, Moustafa Elkalaf, Michal Anděl

**Affiliations:** 1 Laboratory for Metabolism and Bioenergetics, Third Faculty of Medicine, Charles University, Prague, Czech Republic; 2 Centre for Research on Diabetes, Metabolism and Nutrition, Third Faculty of Medicine, Charles University, Prague, Czech Republic; University of Pecs Medical School, Hungary

## Abstract

**Background:**

The lipophilic positively charged moiety of triphenylphosphonium (TPP^+^) has been used to target a range of biologically active compounds including antioxidants, spin-traps and other probes into mitochondria. The moiety itself, while often considered biologically inert, appears to influence mitochondrial metabolism.

**Methodology/Principal Findings:**

We used the Seahorse XF flux analyzer to measure the effect of a range of alkylTPP^+^ on cellular respiration and further analyzed their effect on mitochondrial membrane potential and the activity of respiratory complexes. We found that the ability of alkylTPP^+^ to inhibit the respiratory chain and decrease the mitochondrial membrane potential increases with the length of the alkyl chain suggesting that hydrophobicity is an important determinant of toxicity.

**Conclusions/Significance:**

More hydrophobic TPP^+^ derivatives can be expected to have a negative impact on mitochondrial membrane potential and respiratory chain activity in addition to the effect of the biologically active moiety attached to them. Using shorter linker chains or adding hydrophilic functional groups may provide a means to decrease this negative effect.

## Introduction

Lipophilic cations based on the triphenylphosphonium moiety (TPP^+^) have been widely used to target various biologically active substances such as antioxidants [1–6], spin traps [7–10] or various other chemical probes into mitochondria [11, 12]. The accumulation of TPP^+^ derivatives in mitochondria was first described in 1970 [13, 14]. It relies on the electric potential difference maintained across the inner mitochondrial membrane by the action of the respiratory chain and the fact that this membrane can be permeated by large hydrophobic cations.

Assuming a perfectly Nernstian behaviour, a membrane-permeable cation will accumulate in a negatively charged compartment approximately ten-fold for each 60 mV of potential difference. In the case of TPP^+^ derivatives this ideal behaviour is complicated by the fact that the hydrophobicity of the derivative affects both the extent and the rate of accumulation, more lipophilic derivatives accumulate faster and to higher concentrations than the more hydrophilic ones [15, 16].

The TPP^+^ moiety itself is often assumed not to exhibit any significant biological activity, however, its high affinity for phospholipid membranes [17–19] makes it likely to disrupt membrane integrity [20–22] especially in mitochondria where such compounds accumulate, which could also alter the function of mitochondrial membrane proteins such as complexes of the respiratory chain [23].

It has been previously observed that some TPP^+^ compounds negatively affect mitochondrial and cellular respiration [24–26] and may increase proton leak across the inner mitochondrial membrane, e.g. by enhancing the uncoupling effect of palmitate [27], or anionic protonophores[28]. Most published studies used TPP^+^ derivatives with chemically active moieties [29] making it difficult to separate the effect of the TPP^+^ moiety itself. One notable exception is a recent paper by Reily et al. [30], who studied not only the impact on mitochondrial function of biologically active TPP^+^ compounds MitoQ, MitoTEMPOL and MitoE but also ‘inactive’ alkyl derivatives methyl-, butyl- and decylTPP^+^. Their results from Seahorse measurements of MES-13 cells show a general inhibitory effect of all TPP^+^ derivatives on basal respiration accompanied by signs of mitochondrial uncoupling for decylTPP^+^. This study, while useful in highlighting the significant effects of alkylTPP^+^ on cellular respiration, relied on only one source of data, namely the measurements of oxygen consumption and extracellular acidification. These parameters make it difficult to separate effects on membrane potential and respiratory chain activity.

In the present study we decided to use a range of ‘inactive’ TPP^+^ derivatives, namely alkyltriphenylphosphonium bromide salts, and employ additional assays to study the mechanisms of their toxic effects on mitochondrial respiration. We show a clear negative effect of TPP^+^ derivatives on the respiratory chain complexes, on mitochondrial membrane potential and ATP synthesis. We also provide further support for the previous suggestions that these negative effects increase with increasing hydrohobicity of TPP^+^ compounds.

### Materials

All chemicals were purchased from Sigma-Aldrich unless stated otherwise. Decylubiquinol was prepared by dissolving decylubiquinone in acidified ethanol (pH 4), adding a few grains of sodium borohydride (NaBH_4_) and vortexing until the solution became colourless. Aliquots were stored at −20°C under argon. Ferrocytochrome *c* was freshly prepared by adding few grains of sodium dithionite to 1 mM stock of ferricytochrome *c*.

### Collection of rat tissues

Wistar rats 13–15 weeks old weighing 200–300 gm were obtained from AnLab Ltd., Prague, Czech Republic. Animals were sacrificed by diethylether overdose prior to tissue isolation. We collected both gastrocnemii muscles to prepare a homogenate enriched in the mitochondrial fraction. This was approved by the Committee for the Protection of Laboratory Animals of the Third Faculty of Medicine, Charles University in Prague.

### Cell culture conditions

C2C12 cells were obtained from Sigma-Aldrich and grown in Dulbecco-modified Eagle’s medium (DMEM, Life Technologies) containing 1 g/l d-glucose and supplemented with 10% fetal bovine serum, 100 unit/ml penicillin, 100 *μ*g/ml streptomycin, and 1 mM sodium pyruvate. All cultures were incubated at 37°C in an atmosphere of 95% humidity and 5% CO_2_. Cells were passaged every 48 hours.

### Preparation of muscle homogenate enriched in mitochondrial fraction

We prepared a muscle homogenate by modifying a previously described protocol [31]. A freshly removed rat gastrocnemius was washed three times by ice-cold buffer (250 mM sucrose, 5 mM Tris, 1 mM ethylene glycol-bis(2-aminoethylether)-N,N,N′,N′-tetraacetic acid (EGTA), 0.1% fatty acid free bovine serum albumin (BSA), pH 7.4) then flash frozen in liquid nitrogen, and stored at -80°C. On the preparation day, the visible fat and connected tissue were removed by a scalpel blade, then the muscle was finely dissected into small fragments in a glass dish on ice. The muscle pieces were diluted 1:10 in ice-cold muscle homogenization medium (250 mM sucrose, 20 mM Tris, 40 mM KCl, 2 mM EGTA, pH 7.4) then the suspension was transferred to a glass tube and chopped with an UltraTurrax blender followed by homogenisation in a Dounce homogeniser with a motor-driven Teflon plunger at 500 r.p.m (≈ 10 passes). The homogenate was then centrifuged for 15 min at 600 ×g at 4°C. The supernatant was transferred into new tubes on ice then flash frozen in liquid nitrogen and stored at −80°C. Protein concentration in the homogenate was determined using the bicinchoninic acid (BCA) assay.

### Analysis of metabolism

Cellular respiration was measured using the XF-24 analyzer (Seahorse Bioscience). We performed mitochondrial bioenergetic assays based on published protocols [32]. The XF assay medium (bicarbonate-free modified DMEM, Seahorse Bioscience) was supplemented with 4 mM l-glutamine, 1 mM pyruvate, and 1 g/l d-glucose. The pH was adjusted with 1 M NaOH to 7.4 at 37°C. Cells were seeded at a density of 20,000 cells per well and left overnight to attach and proliferate to obtain a monolayer of cells before measurement. After measuring the basal respiration TPP^+^ derivatives or vehicle were injected and a mitochondrial respiration test was performed by sequential additions of 1 *μ*M oligomycin, 0.5 *μ*M carbonyl cyanide-4-(trifluoromethoxy)phenylhydrazone (FCCP) and 1 *μ*M rotenone and antimycin A. Proton leak-induced respiration was calculated as the difference between respiration achieved after oligomycin addition and non-mitochondrial oxygen consumption following rotenone-antimycin A treatment. Maximal respiration induced by FCCP uncoupler was corrected by subtracting the non-mitochondrial respiration values. For each well the highest measurement value was selected for each type of measurement and compared to the highest reading for the control.

### Analysis of respiratory chain enzymatic activity

Mitochondrial respiratory chain enzymatic activity was assessed in a homogenate prepared from rat skeletal muscle. Prior to enzymatic assays this homogenate was exposed to three cycles of rapid freeze-thawing. We modified previously described protocols to measure the activity of complex I and II [33], complex III [34], and complex IV [35] to fit in a microplate reader.

We wanted to use TPP^+^ concentrations equivalent to those in energised mitochondria exposed to micromolar external concentrations therefore we assumed approximately a 1000-fold accumulation according to the Nernst equation and normal mitochondrial membrane potential. However, high concentrations of decyl- and dodecyl-TPP^+^ (≥ 300 *μ*M) appear to interfere with complex I and II assays due to the formation of a complex with 2,6-dichloroindophenol (DCIP) with a shifted absorbance maximum, and with the complex IV assay as the addition of ferricyanide to the reaction mixture induces the formation of a precipitate complicating the reading of absorbance. Instead of adding 1 mM alkylTPP^+^ derivatives to the complete assay mixture we therefore decided to preincubate the muscle homogenate with the compounds at this concentration and then add the rest of the assay mixture and thus decrease the final concentration of the TPP^+^ to avoid this interference. It is therefore possible that the effects observed in this study underestimate the real effects in intact cells.

#### Complex I

Complex I assay was performed in an assay mixture composed of 25 mM potassium phosphate, 3.5 g/l BSA, 2 mM ethylenediaminetetraacetic acid (EDTA), 60 *μ*M DCIP, 70 *μ*M decylubiquinone, 1 *μ*M antimycin A and 0.2 mM reduced nicotinamide adenine dinucleotide NADH, pH 7.8. Changes in absorbance were followed at 600 nm. Rotenone sensitive activity was calculated by subtracting the activity of wells with 10 *μ*M rotenone.

#### Complex II

Complex II activity was measured in an assay mixture containing 80 mM potassium phosphate, 1 g/l BSA, 2 mM EDTA, 10 mM succinate, 80 *μ*M DCIP, 50 *μ*M decylubiquinone, 1 *μ*M antimycin A and 3 *μ*M rotenone, pH 7.8. Changes in absorbance were followed at 600 nm. Malonate sensitive activity was calculated by subtracting the activity of wells with 20 mM malonate.

#### Complex III

Complex III activity was measured in an assay mixure containing 50 *μ*M ferricytochrome *c*, 25 mM potassium phosphate, 4 mM sodium azide, 0.1 mM EDTA, 0.025% Tween^®^20 and 50 *μ*M decylubiquinol, pH 7.4. Changes in absorbance were followed at 550 nm. Antimycin A sensitive activity was calculated by subtracting the activity of wells with 10 *μ*M antimycin A.

#### Complex IV

Complex IV activity was measured in an assay buffer containing 30 mM potassium phosphate and 25 *μ*M of freshly prepared ferrocytochrome *c*, pH 7.4. Changes in absorbance were followed at 550 nm. The absorbance of samples oxidised with 10 *μ*l of 0.5 M potassium hexacyanoferrate(III) was subtracted from all measurements, then the natural logarithm absorbance was plotted against time and compared to untreated control.

### Detection of changes in mitochondrial membrane potential (Δ*ψ*_*m*_)

Qualitative changes in Δ*ψ*_*m*_ were determined as the changes in tetramethylrhodamine methyl ester (TMRM) fluorescence [36, 37] in non-quench mode [38] in C2C12 myoblasts. Cells were allowed to grow and reach ≈ 80% confluence, then we washed them twice with warm PBS and detached using trypsin for harvesting. The cell suspension was centrifuged at 500 ×g at room temperature for 5 minutes. The pellet was resuspended in warm DMEM containing 50 nM TMRM (Life Technologies) for 20 minutes at 37°C with continuous gentle shaking. The cell suspension was then centrifuged and the pellet was resuspended in warm DMEM and exposed for 10 minutes to tested compounds or vehicle. A FACSCalibur flow cytometer (BD Biosciences) was used to read fluorescence with an excitation wavelength of 488 nm.

### Statistical analyses

Data are presented as mean and 95% confidence interval (CI). One-way ANOVA with Tukey’s multiple comparisons test was performed using GraphPad Prism version 6.0d for Mac OS X, GraphPad Software, La Jolla California USA, www.graphpad.com. Differences found statistically significant are marked with an asterisk. The number of independent experiments is denoted as n.

## Results

### ‘Inactive’ TPP^+^ compounds impair mitochondrial function in intact cells

Firstly we evaluated the effects of TPP^+^ compounds on mitochondrial respiration of intact cells. Basal mitochondrial respiration is controlled by two processes: ATP production and proton leak. We can block ATP synthase by oligomycin, which allows us to measure proton leak-driven respiration. The addition of a highly effective protonophore (FCCP) removes the regulatory effect of the membrane potential and allows us to measure the maximal respiratory rate at a given substrate availability, which will detect any inhibition of the respiratory chain complexes. The extracellular acidification rate (ECAR) is usually interpreted as the production of lactate in glycolysis [32].

**Fig 1:**
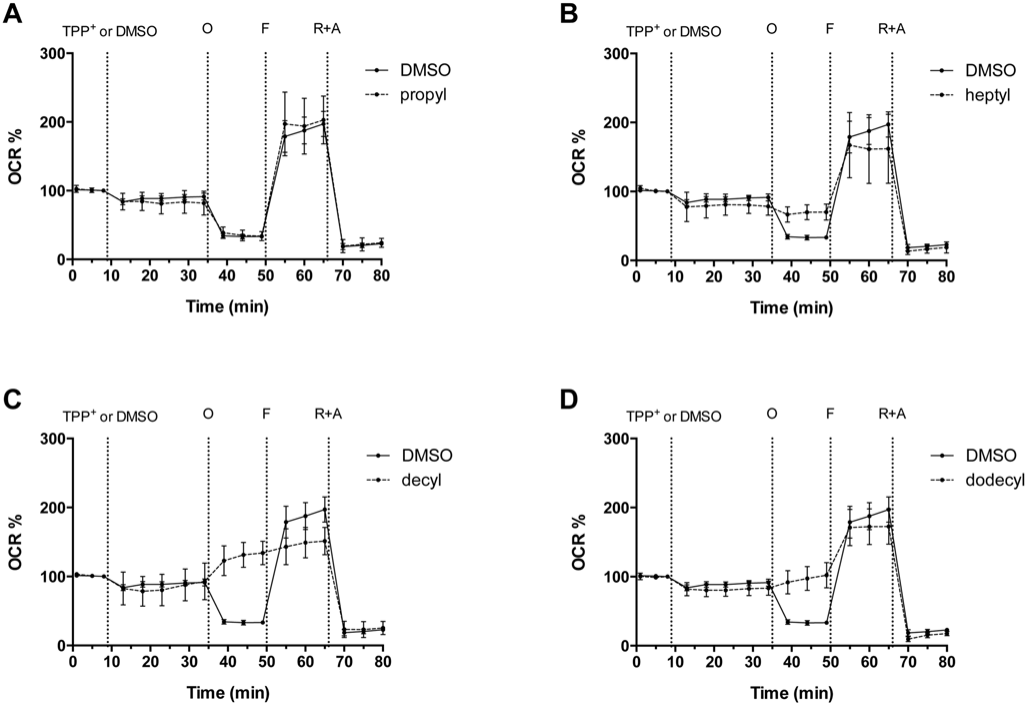
The acute response of cellular respiration to various alkylTPP^+^ compounds in intact C2C12 myoblasts. After measuring the respiration in basal conditions, cells were treated with different alkylTPP^+^ at a final concentration of 1 *μ*M or vehicle (DMSO 0.02%). Oligomycin (O) 1 *μ*M, FCCP (F) 0.5 *μ*M rotenone-antimycin A (R+A) 0.5 *μ*M were injected successively to perform a mitochondrial stress test. Cellular oxygen consumption rate (OCR) is expressed as the percentage of basal OCR (OCR%) and presented as means ± 95% CI, n = 3–6.

**Fig 2:**
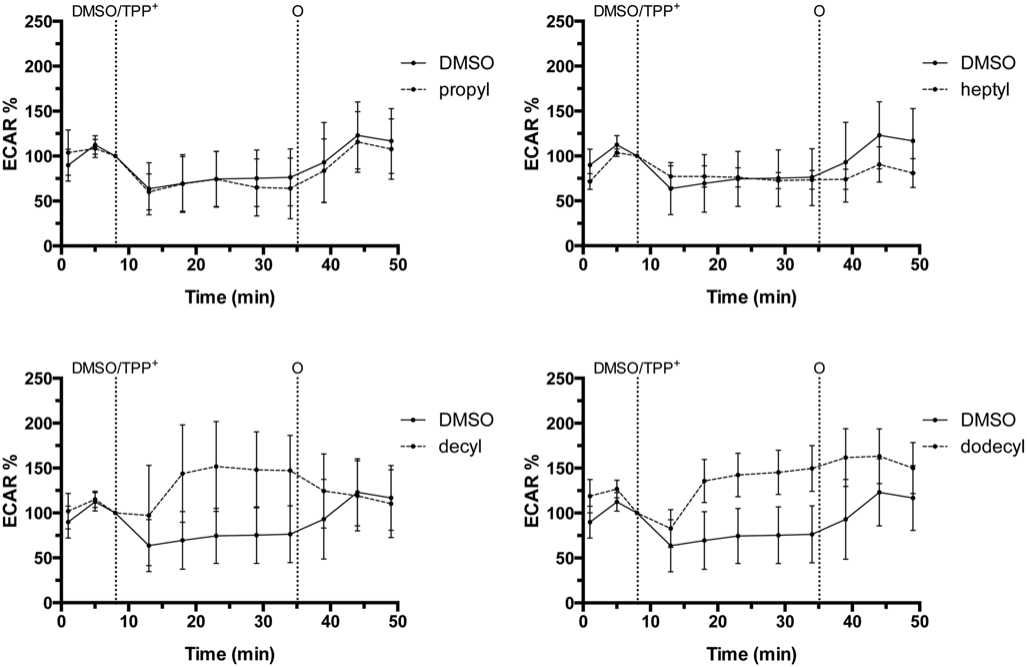
Changes in glycolytic rate in response to various alkylTPP^+^ compounds in intact C2C12 myoblasts. As in Fig 1, cells were treated with different alkylTPP^+^ at a final concentration of 1 *μ*M or vehicle (DMSO 0.02%). The addition of oligomycin (O) stimulates the maximum glycolytic capacity. The extracellular acidification rate (ECAR) is expressed as the percentage of basal ECAR (ECAR%) and presented as means ± 95% CI, n = 3–6.

**Fig 3:**
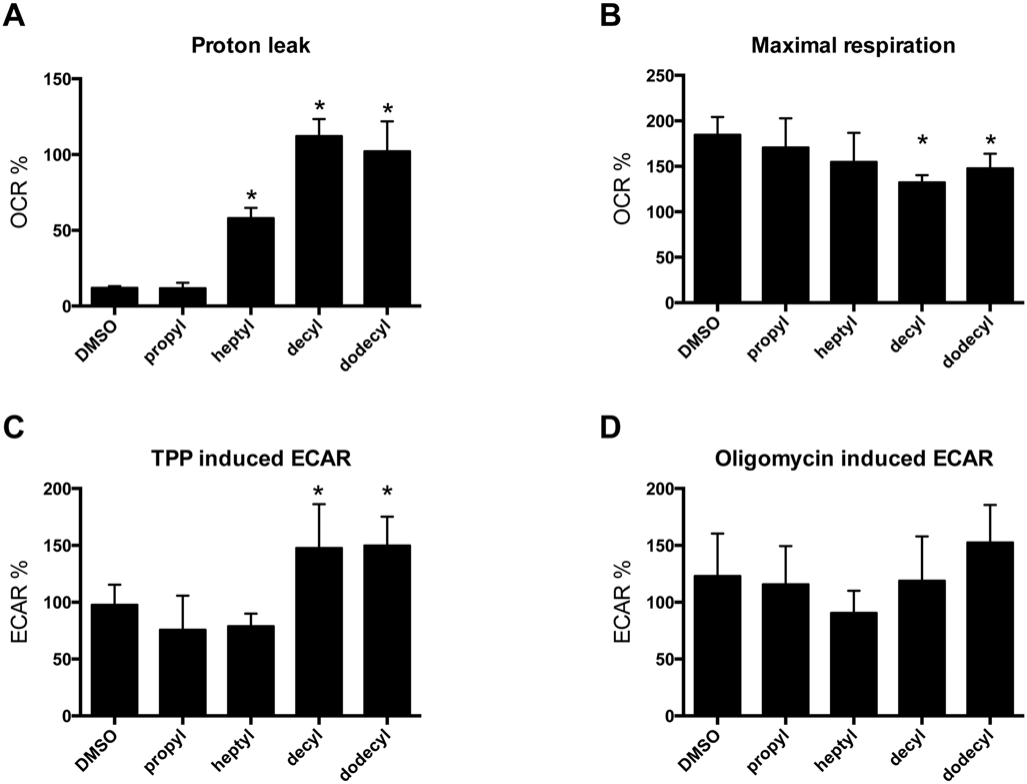
Effect of alkylTPP^+^ compounds (1 *μ*M) on mitochondrial metabolism in intact cells. **A.** Long alkyl chain TPP^+^ derivatives cause an increase in proton leak-driven respiration. **B.** Decyl- and dodecylTPP^+^ cause an inhibition of maximal respiration. **C.** Cells respond to alkylTPP^+^ addition by shifting ATP production to the glycolytic pathway as indicated by an increase in extracellular acidification rate (ECAR). **D.** No significant effect of alkylTPP^+^ compounds on maximum glycolytic capacity induced by oligomycin. All results are expressed as a percentage of basal cellular OCR or basal ECAR and are presented as means ± 95% CI, n = 3–6. * indicates p < 0.05 when compared to the DMSO treated group.

We observed three main effects: the longer-chain alkylTPP^+^ derivatives increased proton leak, decreased maximal respiration (Fig 1) and induced an increase in ECAR (Fig 2). The addition of 1 *μ*M decyl- or dodecylTPP^+^ lead to ≈ 10 fold increase in proton leak-driven (oligomycin-inhibited) respiration, compared to controls treated with the vehicle alone. HeptylTPP^+^ exhibited a more modest effect, while the shortest alkyl derivative, propylTPP^+^ had no effect at this concentration (Fig 3A).

The effect on maximal respiration was somewhat less pronounced at the 1 *μ*M concentration but a similar trend of increasing efficacy with increasing alkyl chain length was apparent (Fig 3B) with only decyl- and dodecylTPP^+^ exhibiting a significant inhibitory effect. Of course, under conditions of complete uncoupling due to FCCP it is likely that the effective concentrations of TPP^+^ derivatives inside mitochondria are much lower than under basal conditions suggesting that the impact of TPP^+^ on the respiratory chain activity could be much larger than shown by these data.

Both increased proton leak and decreased activity of the respiratory chain can be expected to decrease the mitochondrial membrane potential and therefore to have a deleterious effect on mitochondrial ATP synthesis, which may result in a stimulation of glycolysis to make up for ATP deficit. In Fig 3C we show a stimulatory effect of longer chain alkylTPP^+^ derivatives on ECAR, which once again follows the same relationship between alkyl chain length and the effect size. The addition of oligomycin completely blocks oxidative phosphorylation and leads to a further increase of ECAR under basal conditions and we observed no further effects of alkylTPP^+^ derivatives on this rate (Fig 3D).

**Fig 4:**
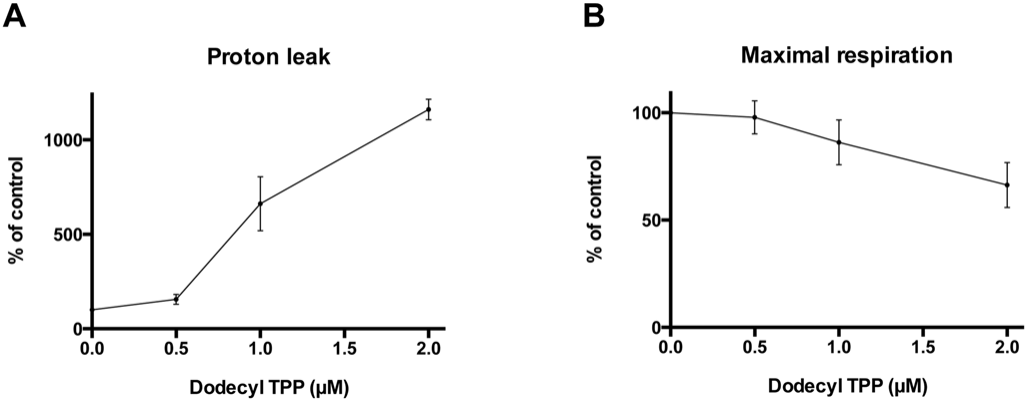
Dose-dependence of the effect of dodecylTPP^+^ on mitochondrial metabolism. **A.** Proton leak-driven respiration increases substantially with an increasing extracellular concentration of dodecylTPP^+^. **B.** A simultaneous decrease in maximal respiration due to increasing doses of dodecylTPP^+^. All results are expressed as the percentage of OCR of the DMSO treated control and are presented as means ± 95% CI, n = 3–6.

We were also interested whether this effect of alkylTPP^+^ derivatives on mitochondrial proton leak and maximal respiration is dose-dependent. Fig 4 shows a clear relationship between both effects and the concentration of dodecylTPP^+^ to which cells were exposed. The proton leak stimulation appears to be much stronger than the inhibitory effect on maximal respiration.

In order to elucidate further these two effects of alkylTPP^+^ derivatives we decided to investigate which respiratory complexes are inhibited by these chemicals, and to measure directly their effect on the mitochondrial membrane potential.

### Inhibition of respiratory chain complexes

We measured the effect of alkylTPP^+^ compounds on the enzymatic activity of individual mitochondrial respiratory chain complexes in freeze-thawed rat skeletal muscle homogenate enriched in the mitochondrial fraction. This model allows a direct access to the respiratory chain in the absence of a mitochondrial membrane potential.

**Fig 5:**
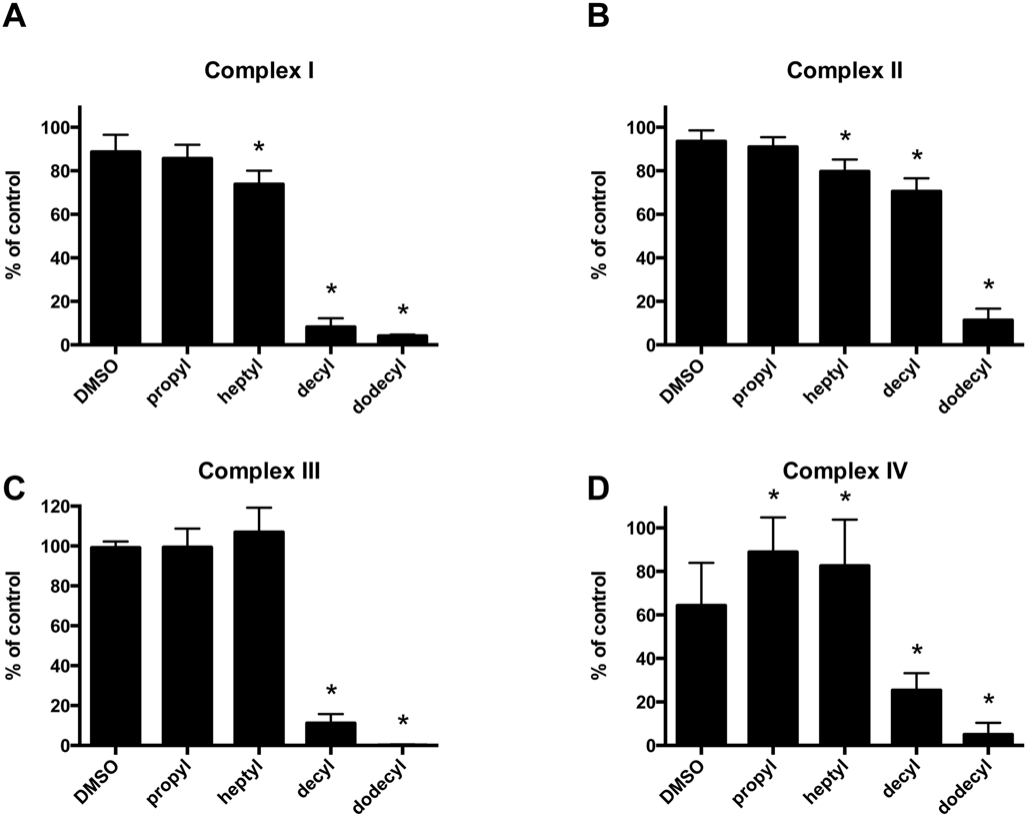
Activity of mitochondrial respiratory chain complexes in rat skeletal muscle homogenate is inhibited by longer-chain alkylTPP^+^ compounds. The homogenate was preincubated with 1 mM TPP^+^ compounds. **A.** Complex I activity was slightly affected by DMSO alone and longer-chain TPP^+^ derivatives caused a marked inhibition. **B.** Complex II activity was less affected with only significant inhibition caused by by dodecylTPP^+^. **C.** Complex III activity was affected in a similar manner as complex I. **D.** DMSO decreased the activity of complex IV by about 40%, and TPP^+^ compounds with shorter chains appear to alleviate this inhibition. Longer chain derivatives, however, caused a marked inhibition of complex IV activity. All results are expressed as the percentage of the activity of the untreated control and are presented as means ± 95% CI, n = 3. * indicates p < 0.05 when compared to the DMSO treated group.

Fig 5 shows a significant inhibitory effect of long alkyl chain TPP^+^ derivatives on all four complexes. Complexes I and III appear to be the most sensitive to TPP^+^ inhibition, while complex II seems to be relatively resistant. The effect of TPP^+^ derivatives on complex IV activity is rather curious. There is a significant inhibitory effect of the vehicle (1% dimethylsulfoxide (DMSO) during preincubation, 0.05% during assay), which is further potentiated by decyl- and dodecylTPP^+^. The shorter chain derivatives, propyl- and heptylTPP^+^, on the other hand, appear to alleviate the toxic effect of DMSO.

**Fig 6:**
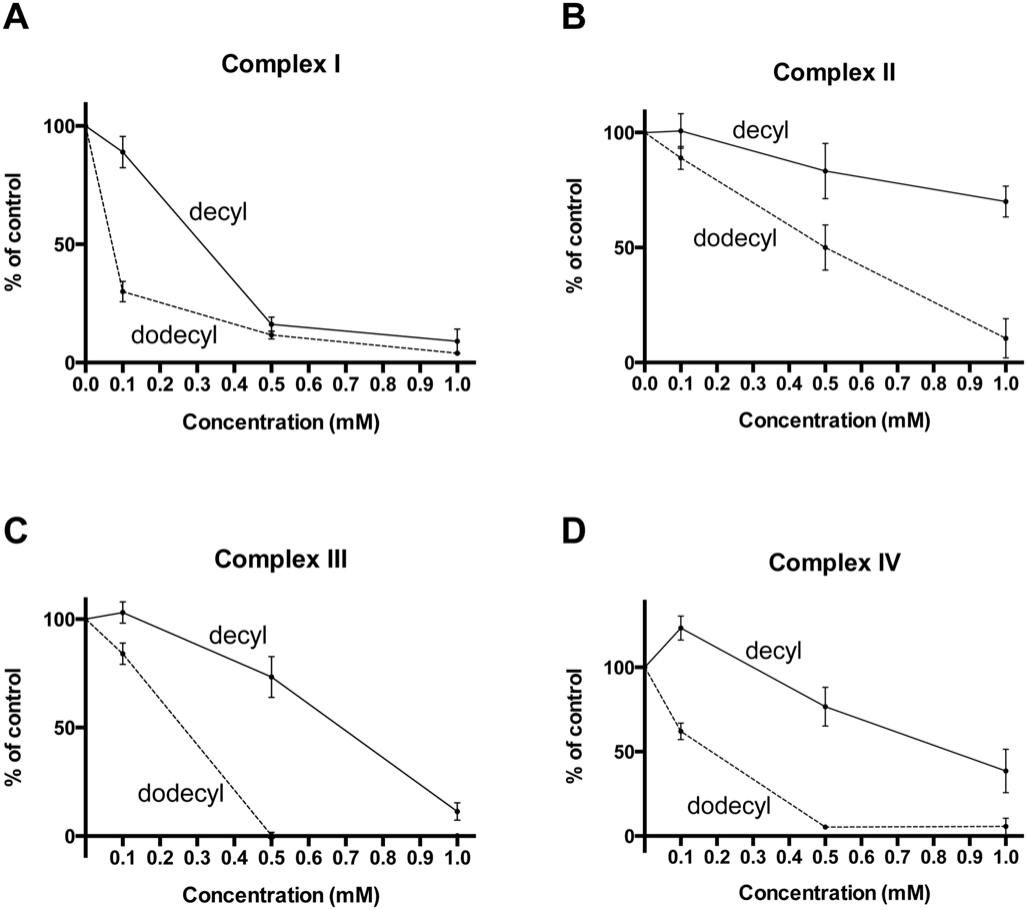
The inhibition of mitochondrial respiratory chain complex activity by longer chain TPP^+^ compounds is dose dependent. Samples of rat skeletal muscle homogenate were incubated with various concentrations of decylTPP^+^ and dodecylTPP^+^ prior to the assay. **A, B, and C.** Complexes I, II, and III show a gradual decrease in activity proportionate with the dose of the TPP^+^ compounds. DodecylTPP^+^ exhibits a stronger inhibitory effect than decylTPP^+^. **D.** A low concentration of decylTPP (100 *μ*M) appears to cause a slight ‘activation’ of complex IV activity, while higher concentrations caused inhibition. DodecyTPP^+^ is once again the more potent inhibitor. All results are expressed as the percentage of the activity of the DMSO treated sample and are presented as means ± 95% CI, n = 3.

For the two longest derivatives we also investigated the dose-dependency of the inhibition of individual complexes (Fig 6). DodecylTPP^+^ virtually completely inhibited complexes I, III and IV at a 0.5 mM concentration, while complex II is only inhibited by about 50% at this concentration. DecylTPP^+^ exhibits a similar pattern with a substantially lower potency.

### TPP^+^ derivatives decrease mitochondrial membrane potential

We sought to strengthen our data on proton leak-driven respiration by using the negatively charged, membrane-permeable fluorescent dye TMRM to estimate the effect of TPP^+^ derivatives on the mitochondrial membrane potential. TMRM accumulates in mitochondria proportionately to the membrane potential and therefore cells with a higher membrane potential will fluoresce with a higher intensity. We detected fluorescence in individual cells using flow cytometry.

**Fig 7:**
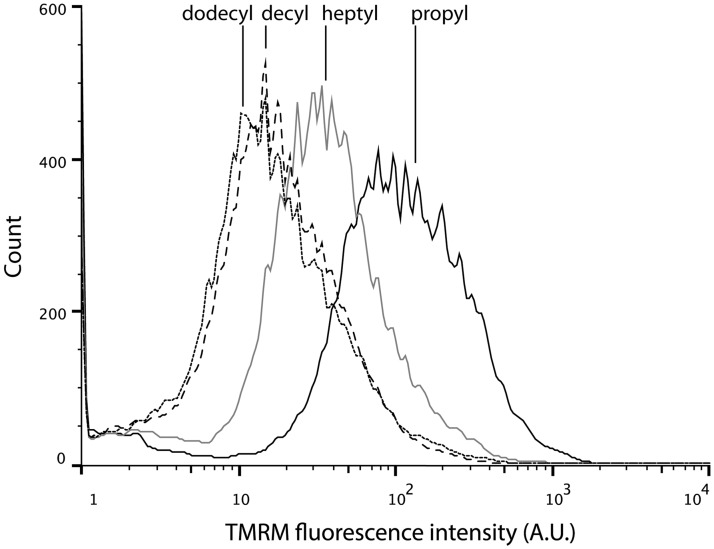
TPP^+^ derivatives decrease mitochondrial membrane potential. A typical TMRM fluorescence intensity histogram from a flow cytometry experiment with C2C12 cells in the presence of 1 *μ*M TPP^+^ compounds. Lower fluorescence intensity corresponds to a lower membrane potential (Δ*ψ*_*m*_).

Fig 7 shows a typical fluorescence intensity histogram and Table 1 summarises the mean fluorescence intensities measured in our experiments. There is a clear trend towards lower fluorescence intensities as the alkyl chain length increases. While 1 *μ*M propyTPP^+^ virtually doesn’t affect the membrane potential heptyl-, decyl- and dodecylTPP^+^ decrease it significantly. Intriguingly, the two longest chain derivatives appear to collapse the mitochondrial membrane potential even more effectively than an equal concentration of the uncoupler FCCP. This could potentially be explained by the combined action of an uncoupling effect and respiratory chain inhibition.

**Table 1:** Mean fluorescence intensity of TMRM in C2C12 cells treated with 1 *μ*M TPP^+^ derivatives.

**Treatment**	**Fluorescence intensity (% of untreated control)**
DMSO	94,50 [87.56,101.4]
propylTPP^+^	93,75 [88.18,99.32]
heptylTPP^+^	35,50 [25.07,45.93]*
decylTPP^+^	19,25 [2.39,36.11]*
dodecylTPP^+^	18,00 [0.71,35.29]*
FCCP	43,50 [25.47,61.53]*

Data are geometrical means of fluorescence intensity expressed as the percentage of the untreated control [95% CI], n = 4.

* indicates p < 0.05 when compared to the DMSO treated group.

## Discussion

The TPP^+^ moiety of mitochondrially targeted compounds is often considered to be without a significant biological activity. Here we show a clear evidence that TPP^+^ derivatives with simple alkyl chains in place of ‘active’ chemical moieties may significantly affect mitochondrial bioenergetics.

In particular, we observed a significant potentiation of proton leak with an ensuing decrease in the mitochondrial membrane potential and an inhibition of the respiratory chain complexes. Our expectation that the magnitude of these effects may correlate with the alkyl chain length and therefore hydrophobicity of the compounds was also supported by the data.

TPP^+^ compounds are well known to have a high affinity to biological membranes [39]. This affinity will further increase with an increasing hydrophobicity of the derivative [15, 16]. A plausible explanation of our observations therefore may be that both the increase in proton leak and the inhibition of respiratory chain complexes is mediated by an incorporation of alkylTPP^+^ molecules into the inner mitochondrial membrane and the resulting disruption of its normal function. Since the respiratory complexes are known to be sensitive to their lipid environment and require phospholipid molecules for their activity [40–42], a high proportion of alkylTPP^+^ molecules in the membrane could impair both the insulant properties of the membrane allowing protons to leak back into the matrix and the membrane structure required for the functioning of the protein complexes.

Data from intact cells presented in this work or previously published [30] are useful to identify broad effects on mitochondrial bioenergetics but additional assays are required to pinpoint more precise mechanisms of effect of TPP^+^ derivatives. Spectrophotometric assays of individual respiratory chain complexes in tissue homogenate enriched in mitochondrial fraction allowed us to confirm direct inhibition of all complexes by the longer-chain derivates with complex II being the least sensitive and establish dose-dependency of these effects. We also provide independent determination of a negative effect on the mitochondrial membrane potential.

The exact mechanism of respiratory chain inhibition can only be speculated about based on our data. The fact that the inhibitory effect is not specific to any one derivative or any one complex suggests a non-specific binding of the TPP^+^ derivates to the inner mitochondrial membrane, which affects membrane integrity causing both the breakdown of its insulating properties and impairment of the phospholipid milieu faced by the respiratory complexes.

In summary, TPP^+^ derivatives impair mitochondrial function with an increasing potency as their hydrophobicity increases. This may help explain some effects of existing mitochondrially targeted compounds and should be taken into account when designing new ones for use as diagnostic probes or therapeutic agents.
